# Mechanisms, Biomarkers, and Therapeutics Involved in Inflammatory Disorders and Tissue Repair 2021

**DOI:** 10.1155/2022/9806128

**Published:** 2022-03-21

**Authors:** Reggiani Vilela Gonçalves, Mariáurea Matias Sarandy, Débora Esposito, Maria do Carmo Gouveia Peluzio

**Affiliations:** ^1^Animal Biology Department, Federal University of Viçosa, Viçosa, 36570-000 Minas Gerais, Brazil; ^2^Animal Biology and Cellular and Structural Biology Graduate Program, Federal University of Viçosa, Viçosa, 36570-000 Minas Gerais, Brazil; ^3^Department of Animal Science, Regenerative Medicine, North Carolina State University, 28081 NC, USA; ^4^Department of Nutrition and Health, Federal University of Viçosa, Viçosa, 36570-000 Minas Gerais, Brazil

The inflammation during wound healing process is characterized by activation of cellular, vascular, and biochemical mechanisms that occur to perform a fast tissue repair and avoid complications [1, 2]. The first cells to migrate to the injured area are neutrophils, which recruit macrophages by chemotaxis. These cells promote the release of cytokines, growth factors, and reactive oxygen species (ROS) and nitrogen (RNS) responsible for a good evolution of the wound healing process [3]. The excess of ROS and RNS generated during inflammation can lead to cell damage, such as membrane disorganization and protein oxidation, altering cell functions [4, 5]. Furthermore, the excess of proinflammatory mediators promotes an increase in the content of hydrogen peroxide (H_2_O_2_) and nitric oxide, which accelerates the peroxidation of cell components [6, 7]. The balance between ROS production and antioxidant defenses is important for the resolution of inflammatory diseases as well as for efficient tissue repair [4, 5, 6]. Therefore, a controlled inflammation process is necessary to avoid persistent tissue damage through the continuous action of free radicals, ROS, and RNS [7].

Cytokines, growth factors, oxidative markers, and other molecular markers are considered biomarkers that allow understanding the progression of inflammatory disorders and tissue repair processes. Currently, different therapeutic interventions and their mechanism of action (cellular and extracellular) have been investigated to better understand this complex process [8]. In this context, several interventions have been investigated for the treatment of inflammatory diseases, and in this edition, we observed the high potential of these compounds for the treatment of inflammatory diseases (e.g., melatonin; Hibiscus sabdariffa; atorvastatin; olaparib, curcumin, and plant extracts in general) exhibiting antioxidant and anti-inflammatory actions. In many cases, these properties are based on the interruption of cellular redox metabolism, suppressing oxidative stress, a pharmacological effect that opens new spaces for the reuse of drugs, and the development of new strategies for the treatment of inflammatory disorders and tissue repair. Furthermore, the anti-inflammatory action of different interventions has been attributed to modulation of the STAT-3, AP-1, NFkB, and Nrf2 pathways.

This special issue brings together a set of thirteen studies in an interdisciplinary platform that addresses the inflammatory subcellular, cellular, and molecular bases associated with redox metabolism. This special issue also highlights the ongoing effort to understand the redox systems associated with inflammatory disorders and tissue repair at all levels and to understand how the action of different treatments can lead to reduced gene expression of inflammatory proteins and increased levels of antioxidant enzymes. Thus, this issue contains seven articles that describe different mechanisms involved in this complex process, under different pathological conditions, which are briefly mentioned in the following.

Some studies have shown that external agents such as food, lack of sleep, and physical activity reflect an imbalance between oxidant and antioxidant mechanisms in favor of oxidants, in addition to inhibiting anti-inflammatory pathways, thus causing tissue damage. These prooxidant mechanisms are related to genetic and epigenetic regulation and can regulate the expression of molecules that can activate the signal transduction pathways responsible for inflammation and cause oxidative damage to proteins, lipids, and DNA. Thus, antioxidant systems seem to play a crucial role in maintaining tissue morphological and functional integrity. Phenolic compounds present in herbal medicines, in addition to oncological drugs, drugs of the statin class, and hormones, have shown efficacy in inflammatory disorders and intestinal preneoplastic lesions, testicular, oral mucositis, and muscle inflammatory damage by suppressing oxidative stress, attenuating cellular responses, activating intracellular pathways, and reducing cell death.

Ideally, inflammatory modulators should be able to reduce proinflammatory cytokines and increase anti-inflammatory cytokines and antioxidant defenses. Furthermore, it is important to emphasize that chronic inflammation associated with high oxidative stress can cause organelle damage, leading to a lot of diseases that compromise the good function of the cells. In this context, we can highlight that Hibiscus sabdariffa reduces intestinal preneoplastic lesions in a murine model, attenuating inflammation cellular responses in the initial phase of these lesions. On the other hand, curcumin associated with physical activity reduces muscle damage as well as proinflammatory markers. In contrast, the increase in oxidative stress promoted by plant extracts used with sex inhibitors was reported in a systematic review of this special issue. Furthermore, high levels of inflammatory cytokines were found in patients with exotropia, associating this condition with the inflammatory process. The hormone melatonin reduced colitis and restored the intestinal microbiota by reducing oxidative stress and the inflammatory response.

We hope that the readers of this special issue will find these findings interesting and useful to advance the understanding of such a complex and multifaceted theme, suggesting update for this interesting topic.
